# A self-supervised vision transformer to predict survival from histopathology in renal cell carcinoma

**DOI:** 10.1007/s00345-023-04489-7

**Published:** 2023-06-29

**Authors:** Frederik Wessels, Max Schmitt, Eva Krieghoff-Henning, Malin Nientiedt, Frank Waldbillig, Manuel Neuberger, Maximilian C. Kriegmair, Karl-Friedrich Kowalewski, Thomas S. Worst, Matthias Steeg, Zoran V. Popovic, Timo Gaiser, Christof von Kalle, Jochen S. Utikal, Stefan Fröhling, Maurice S. Michel, Philipp Nuhn, Titus J. Brinker

**Affiliations:** 1 Digital Biomarkers for Oncology Group, National Centre for Tumour Diseases (NCT), German Cancer Research Centre (DKFZ), Im Neuenheimer Feld 280, 69120 Heidelberg, Germany; 2 Department of Urology and Urological Surgery, Medical Faculty Mannheim of Heidelberg University, University Medical Centre Mannheim, Theodor-Kutzer-Ufer 1-3, 68167 Mannheim, Germany; 3 Institute of Pathology, Medical Faculty Mannheim of Heidelberg University, University Medical Centre Mannheim, Theodor-Kutzer-Ufer 1-3, 68167 Mannheim, Germany; 4 Department of Clinical-Translational Sciences, Berlin Institute of Health (BIH), Charité University Medicine, Berlin, Germany; 5 Skin Cancer Unit, German Cancer Research Centre (DKFZ), Heidelberg, Germany; 6 Department of Dermatology, Venereology and Allergology, University Medical Centre Mannheim, University of Heidelberg, Heidelberg, Germany; 7 National Centre for Tumour Diseases, German Cancer Research Centre, Heidelberg, Germany

**Keywords:** Artificial intelligence, Deep learning, Kidney neoplasms, Treatment outcome, Risk assessment, Oncology, Survival analysis

## Abstract

**Purpose:**

To develop and validate an interpretable deep learning model to predict overall and disease-specific survival (OS/DSS) in clear cell renal cell carcinoma (ccRCC).

**Methods:**

Digitised haematoxylin and eosin-stained slides from The Cancer Genome Atlas were used as a training set for a vision transformer (ViT) to extract image features with a self-supervised model called DINO (self-distillation with no labels). Extracted features were used in Cox regression models to prognosticate OS and DSS. Kaplan–Meier for univariable evaluation and Cox regression analyses for multivariable evaluation of the DINO-ViT risk groups were performed for prediction of OS and DSS. For validation, a cohort from a tertiary care centre was used.

**Results:**

A significant risk stratification was achieved in univariable analysis for OS and DSS in the training (*n* = 443, log rank test, *p* < 0.01) and validation set (*n* = 266, *p* < 0.01). In multivariable analysis, including age, metastatic status, tumour size and grading, the DINO-ViT risk stratification was a significant predictor for OS (hazard ratio [HR] 3.03; 95%-confidence interval [95%-CI] 2.11–4.35; *p* < 0.01) and DSS (HR 4.90; 95%-CI 2.78–8.64; *p* < 0.01) in the training set but only for DSS in the validation set (HR 2.31; 95%-CI 1.15–4.65; *p* = 0.02). DINO-ViT visualisation showed that features were mainly extracted from nuclei, cytoplasm, and peritumoural stroma, demonstrating good interpretability.

**Conclusion:**

The DINO-ViT can identify high-risk patients using histological images of ccRCC. This model might improve individual risk-adapted renal cancer therapy in the future.

## Introduction

Clear cell RCC (ccRCC) shows the worst prognosis after surgery of the three most common RCC histological subtypes (papillary, chromophobe and ccRCC) with an estimated 5-year survival rate of 75% [1].

The identification and evaluation of biomarkers for better risk stratification within the subtypes is an ongoing challenge [2–4]. Recently, artificial intelligence (AI)-based image analysis of haematoxylin and eosin-stained (H&E) histopathological tissue sections has demonstrated potential as a low-cost method to predict genetic mutations and other relevant alterations in oncology, including genitourinary tumours [5–7]. Therefore, AI has gained popularity in biomarker research.

One major drawback in the development of many AI models has been the need to provide the model with labelled data. Newer approaches aim for models that can be trained on unlabelled data such as self-supervised learning models. When applied to image recognition, such model is tasked to identify image features that serve as meaningful representation for the images provided in the dataset. Especially in medical image recognition, where labelled images are scarce, this method can help to build potentially more accurate and generalisable models.

Vision transformer (ViT) is a transparent deep learning approach. In contrast to many other deep learning models, ViT uses the position of the different objects and their relationship to each other. Furthermore, ViT makes use of the concept of “attention” by merging input from multiple “attention heads” that focus on different image structures. Visualisation of the “attention” structures provides a high level of transparency. Thus, ViTs are increasingly used in medical research [8–10].

In a recent work, a self-supervised model called DINO (self-distillation with no labels) was combined with a ViT [11]. This combination was designed to identify recurring structures on the images independent of image labels, for example resulting in the identification of different animals independent of the background [11].

The application of such models in outcome prediction in ccRCC has not been investigated. We thus made use of the combination of a ViT and DINO (DINO-ViT) to extract image features and use the resulting feature vector in a Cox regression model to predict overall and disease-specific survival (OS/DSS) directly from H&E histopathological images in ccRCC and validate this method on unseen data using an external dataset.

## Materials and methods

### Study population

The framework and reporting of this study were designed on the basis of the TRIPOD checklist [12]. For patient/slide inclusion, the ccRCC cohort of The Cancer Genome Atlas (TCGA-KIRC) (training) and patients from the University Medical Centre Mannheim (validation) who had undergone partial or radical nephrectomy between 2006 and 2011 were screened. The following inclusion criteria were applied for the selection:

- Histologically confirmed diagnosis of ccRCC
- Availability of a diagnostic H&E-stained slide of the primary carcinoma used for routine diagnosis
- Information on survival status and survival/follow-up time

Patients/H&E slides were excluded for the following reasons:

- H&E slide containing < 250 patches of ccRCC tissue of sufficient quality

Since no direct information on DSS is available for the TCGA cohort, this information was obtained from a work that developed a standardized data set for DSS using an approximation for the TCGA KIRC cohort [13]. For the validation set, data for the type of death were obtained. Missing data on DSS were only considered an exclusion criterion for the DSS analysis and not for the OS analysis.

This study was approved by the local ethics committee (#2021-862-AF 11). Informed consent was waived for this retrospective analysis.

### Study design

**Fig. 1:**
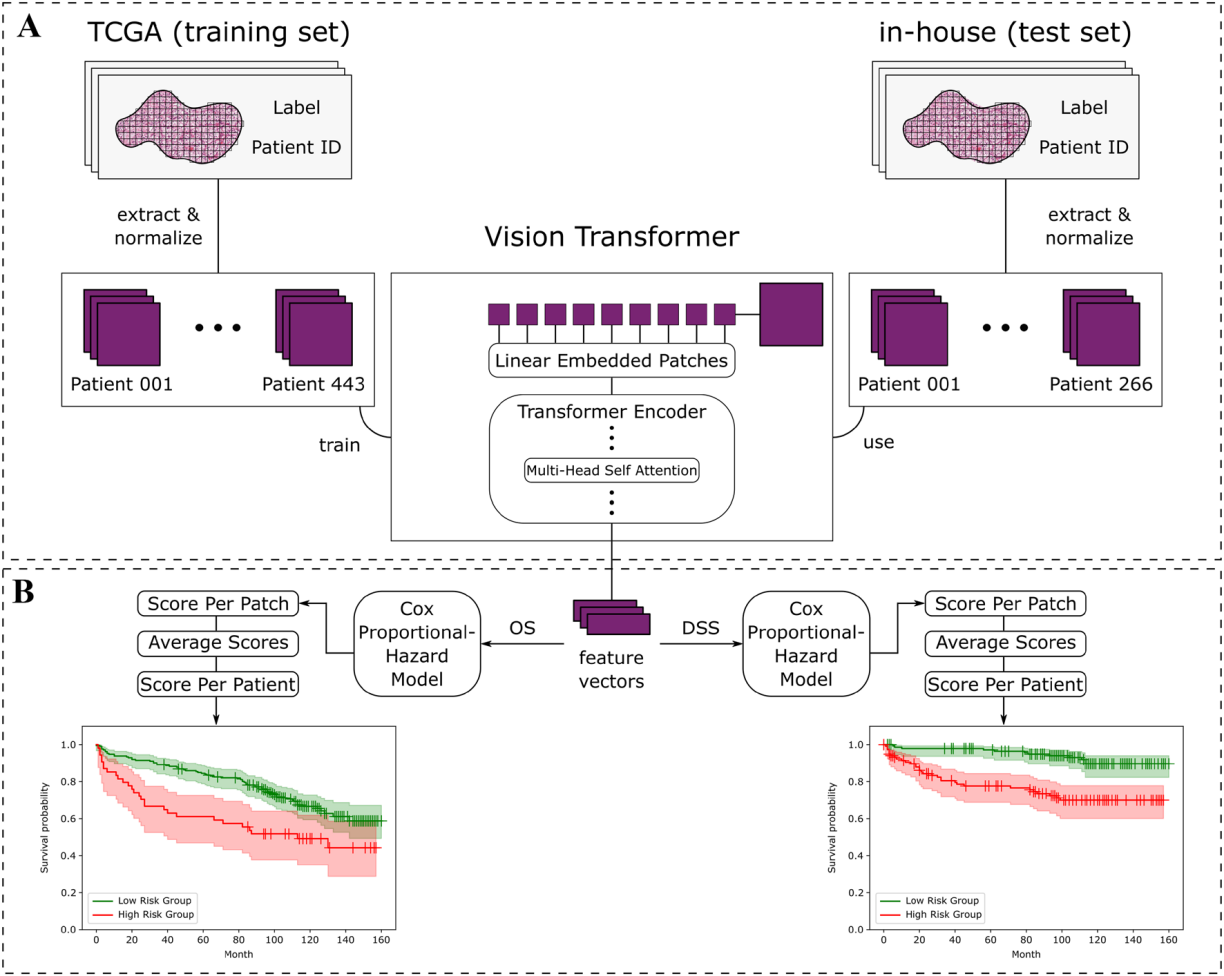
Workflow and study design. **A** The pre-processing of the included slides is shown. For each slide of the TCGA training and in-house test sets, patches were extracted from annotated tumour regions. The patches were used as input for the ViT which extracts image features resulting in a feature vector of each patch. This was done in an unsupervised manner, meaning no labels were provided in the training of the ViT. **B** The resulting feature vectors were used as input for the Cox-Hazard regression model. One model for each endpoint (OS and DSS) was trained, again using the TCGA cohort and then tested on the in-house cohort. For the slide-level prediction, the score per patch was averaged to calculate an average slide score

As depicted in Fig. 1, the basic principle of our method is to train the DINO-ViT model to extract feature vectors from the images (Fig. 1A) followed by training a Cox regression model using the extracted feature vectors for prediction of OS and DSS (Fig. 1B), resulting in a low- and high-risk stratification. The trained DINO-ViT and Cox regression model was then externally validated using a set of our institution (in-house).

#### Pre-processing

Slides from the Mannheim cohort were digitised with 40-fold magnification using a Leica Aperio AT2 DX scanner, resulting in a whole slide images (WSIs) resolution of 0.25 µm/px. WSIs from the TCGA-KIRC cohort were downloaded from the Genomic Data Commons (GDC) data portal. For every WSI, tumour regions were annotated under expert pathologists’ supervision (TG, ZP) and subsequently tessellated into downscaled square patches of 512px x 512px using QuPath 0.2.3 [14]. The Macenko method was used for normalising variances in staining colours [15]. Additionally, blur-detection was implemented in Python version 3.7.7.

#### *Extracting* feature vectors* using a DINO *self-supervised* ViT*

DINO is a newly developed self-supervised learning method [11]. The uniqueness of DINO lies in the use of different image transformations by applying techniques such as cropping and performing the self-supervised learning process from these different presentations of the same image for all images in the dataset. This has proven to achieve more robust underlying features DINO uses to represent the dataset. In this study, the dataset consisted of histological images of ccRCC. Thus, practically, the model was tasked to find recurring structures in the histological images that define ccRCC. Since these structures usually have different individual morphological manifestations which can be extracted by the model using a feature extractor, the output of this architecture, a feature vector, should represent the histological variety of ccRCC. Similar to the original publication, a ViT was used as a backbone for the DINO self-supervised learning model. Using this architecture, the model can segment the images and define boundaries. This information is stored and can be visualised in the built-in attention heads. Attention is a mechanism that allows the model to selectively focus on certain parts of the input image. Each attention head is responsible for computing a different type of attention, in our model translating to focusing on different parts of the image that the model considers to be of importance for the dataset. This Self-attention was thus used to attach weight to each region of an image and adjust the feature extraction accordingly (Fig. 1A), a process that is called attention learning as part of the training of the model.

The model was trained for 300 epochs and used as a self-supervised feature extractor. For each epoch, the model used 1000 randomly sampled patches per WSI. During this process no label was provided. Finally, the model was used to extract a feature vector (384 × 1) for every patch from both the TCGA-KIRC and Mannheim cohorts.

#### Cox regression* model*

For the survival analysis, the learned representations of each histological image, more specifically of every patch, were used in the form of a feature vector, the output of the DINO-ViT model as described above. A Cox proportional hazard model was fitted to predict the endpoints OS and DSS using these feature vectors of every patch per WSI from the TCGA-KIRC cohort. A cross-validation was conducted to determine the value of the parameter “penalizer” based on the highest concordance index. The l1_ratio was set to 1, resulting in a “Least Absolute Shrinkage and Selection Operator” (LASSO) regression. The model was fitted for the TCGA-KIRC cohort and used to predict a risk score for every patch of a WSI. Subsequently, all patches of the same WSI were averaged, resulting in a slide level prediction (Fig. 1B). The risk score represents the time to death. The model was trained using the TCGA cohort. The median of the risk scores of all images in the training set was used as a threshold to define low- and high-risk groups. The trained model was first evaluated on the TCGA training cohort and secondly externally validated on unseen data using the validation set from our institution. The median risk score was again used as threshold for the stratification into low- and high-risk groups.

#### Statistics

A Kaplan–Meier estimator was used to calculate the survival function for both risk groups. A log-rank test determined the difference between both groups. This was done on the TCGA training set and the external validation set after the training process was completed. To evaluate the significance and clinical relevance of the DINO-ViT-based survival prediction, it was compared with already known prognostically relevant clinicopathological parameters using multivariable Cox hazard models. Hazard ratios, confidence intervals and *p* values were calculated for each parameter included in the multivariable prediction of OS and DSS. The included clinical variables were age, grading (G1/G2 vs. G3/G4), tumour stage (T1/T2 vs. T3/T4) and metastasis status (M+ vs. M–). Calculations were performed using JMP 15.2.1 (SAS Institute, Cary, NC, USA).

## Results

### Patient population

709 patients, with one corresponding WSI each, were included in this study. For DSS, *n* = 7 patients were not included in the training TCGA set since no information on DSS was available. Detailed patient characteristics are shown in Table 1.

**Table 1:** Patient cohorts

Variable	TCGA	Mannheim validation
Patients (*n*)	443	266
Age (years)		
Median, IQR	61 (52–69)	64 (56–71)
Sex		
Male, *n* (%)	287 (65)	186 (70)
Tumour size		
pT1, *n* (%)	220 (50)	157 (59)
pT2, *n* (%)	61 (14)	26 (10)
pT3, *n* (%)	151 (34)	79 (30)
pT4, *n* (%)	11 (2)	4 (1)
Grading		
G1, *n* (%)	10 (2)	39 (15)
G2, *n* (%)	181 (41)	207 (77)
G3, *n* (%)	177 (40)	20 (7)
G4, *n* (%)	72 (16)	0 (0)
GX, *n* (%)	3 (1)	2 (1)
Metastasis,		
M+, *n* (%)	70 (16)	26 (10)
Follow-up		
Median follow-up, IQR (months)	37 (17–62)	108 (78–124)
Deaths during follow-up, *n* (%)	155 (35)	99 (37)

*G* grading, *IQR* interquartile range, *M* metastasis, *n* number, *TCGA* The Cancer Genome Atlas

### DINO-ViT performance

**Fig. 2:**
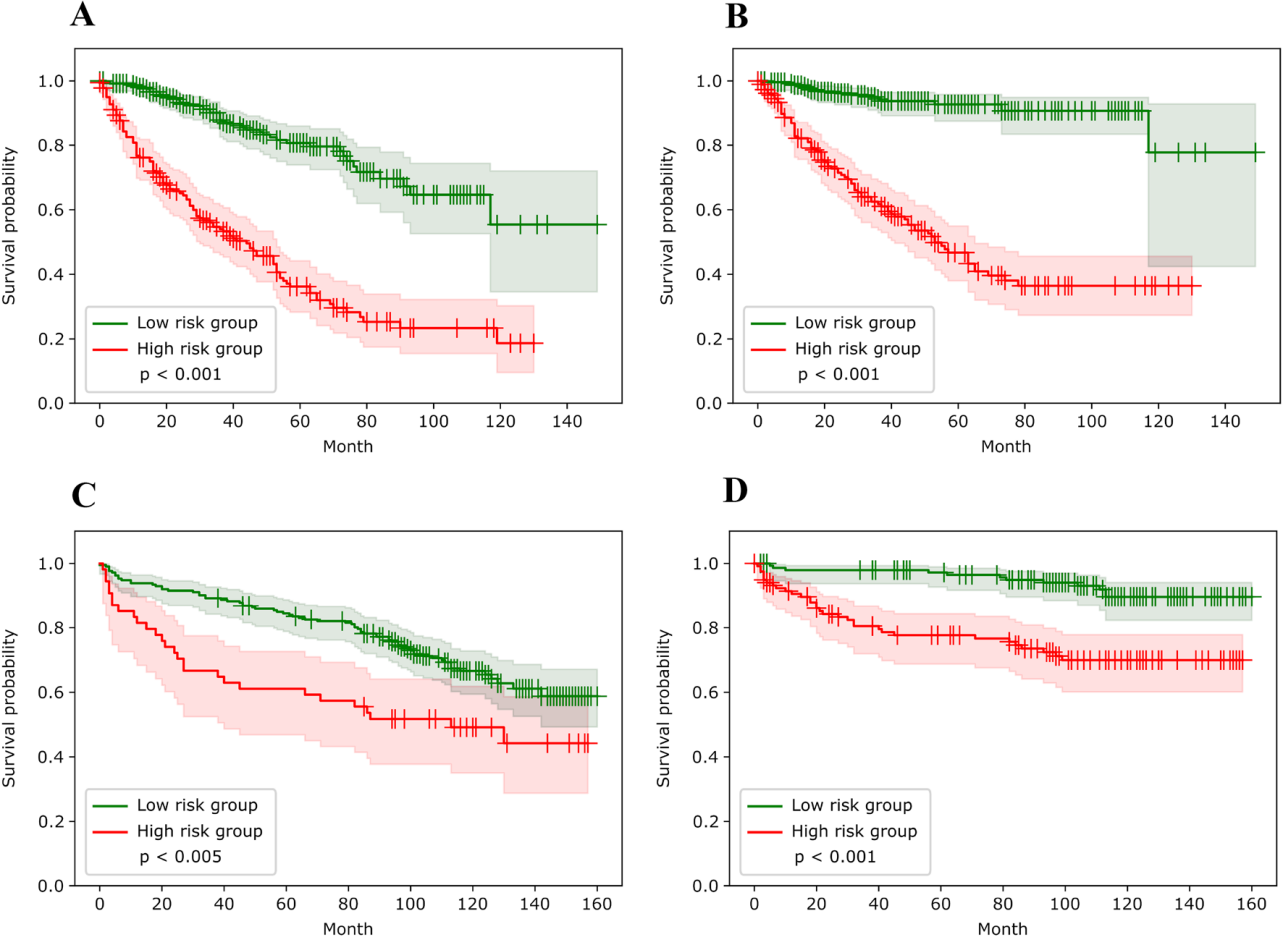
Kaplan–Meier analysis for OS and DSS in the training and validation sets. **A** Kaplan–Meier curve and log-rank test for low- (green) and high-risk (red) groups for OS in the TCGA training set. **B** Kaplan–Meier curve and log-rank test for low- (green) and high-risk (red) groups for DSS in the TCGA training set. **C** Kaplan–Meier curve and log-rank test for low- (green) and high-risk (red) groups for OS in the Mannheim validation set. **D** Kaplan–Meier curve and log-rank test for low- (green) and high-risk (red) groups for DSS in the Mannheim validation set. *DSS* disease-specific survival, *OS* overall survival, *TCGA* The Cancer Genome Atlas

The ViT low-risk group showed a significantly longer OS compared to the high-risk group in the training set (log rank test: *p* < 0.001). As shown in Fig. 2B, DSS in the low-risk group was also significantly longer (*p* < 0.001). In the validation set, there was a significant difference between groups in OS (*p* < 0.005; Fig. 2C) and DSS (*p* < 0.001; Fig. 2D).

### Subgroup analysis

The established DINO-ViT risk stratification was also examined in patients with metastases only. With the threshold defined in the training process for the entire cohort, a significant difference in the Kaplan–Meier analysis could also be demonstrated for OS (log rank test: *p* < 0.01) and DSS (*p* = 0.03) in the TCGA metastatic subgroup. In the small subgroup of the validation set, no significant difference was found for OS (*p* = 0.26) and DSS (*p* = 0.065).

### Multivariable analysis

The DINO-ViT risk group was an independent predictor of OS in the training set (hazard ratio [HR] 3.03; 95%-confidence interval [95%-CI] 2.11–4.35; *p* < 0.01) but not in the validation set (HR 1.25; 95%-CI 0.76–2.06; *p* = 0.38; Table 2). Independent predictors in the validation set were age, grading, TNM tumour size, and the occurrence of metastasis. DINO-ViT was an independent predictor of DSS in the training (HR 4.90; 95%-CI 2.78–8.64; *p* < 0.01) and validation (HR 2.31; 95%-CI 1.15–4.65; *p* = 0.02) sets. Again, grading, tumour size, and metastasis were significant predictors of DSS.

**Table 2:** Multivariable OS and DSS Cox Hazard model

	Hazard ratio	95%-CI	*p* value
**Overall survival**			
* Training set*			
Age (continuous)	1.03	1.01–1.04	< 0.01
Grading (G3/G4 vs. G1/G2)	1.42	0.96–2.09	0.08
Tumour size (T3/T4 vs. T1/T2)	1.41	0.97–2.07	0.07
Metastasis (M+ vs. M–)	2.69	1.85–3.92	< 0.01
DINO-ViT risk group (High vs. Low)	3.03	2.11–4.35	< 0.01
* Validation set*			
Age (continuous)	1.04	1.02–1.06	< 0.01
Grading (G3/G4 vs. G1/G2)	3.41	1.79–6.50	< 0.01
Tumour size (T3/T4 vs. T1/T2)	1.76	1.14–2.74	0.01
Metastasis (M+ vs. M–)	4.22	2.44–7.29	< 0.01
DINO-ViT risk group (High vs. Low)	1.25	0.76–2.06	0.38
**Disease specific survival**			
*Training set*			
Age (continuous)	1.01	0.99–1.03	0.40
Grading (G3/G4 vs. G1/G2)	2.14	1.22–3.76	0.01
Tumour size (T3/T4 vs. T1/T2)	1.61	0.98–2.65	0.06
Metastasis (M+ vs. M–)	4.01	2.57–6.24	< 0.01
DINO-ViT risk group (High vs. Low)	4.90	2.78–8.64	< 0.01
*Validation set*			
Age (continuous)	0.98	0.95–1.01	0.22
Grading (G3/G4 vs. G1/G2)	3.70	1.71–7.99	< 0.01
Tumour size (T3/T4 vs. T1/T2)	3.48	1.72–7.05	< 0.01
Metastasis (M+ vs. M–)	6.5	3.32–12.75	< 0.01
DINO-ViT risk group (High vs. Low)	2.31	1.15–4.65	0.02

*95%-CI* 95% confidence interval, *DINO* self-distillation with no labels, *DSS* disease-specific survival, *G* grading, *M* metastasis, *OS* overall survival, *TCGA* The Cancer Genome Atlas, *ViT* vision transformer

### *Visualis*ation of* the DINO-ViT attention heads*

**Fig. 3:**
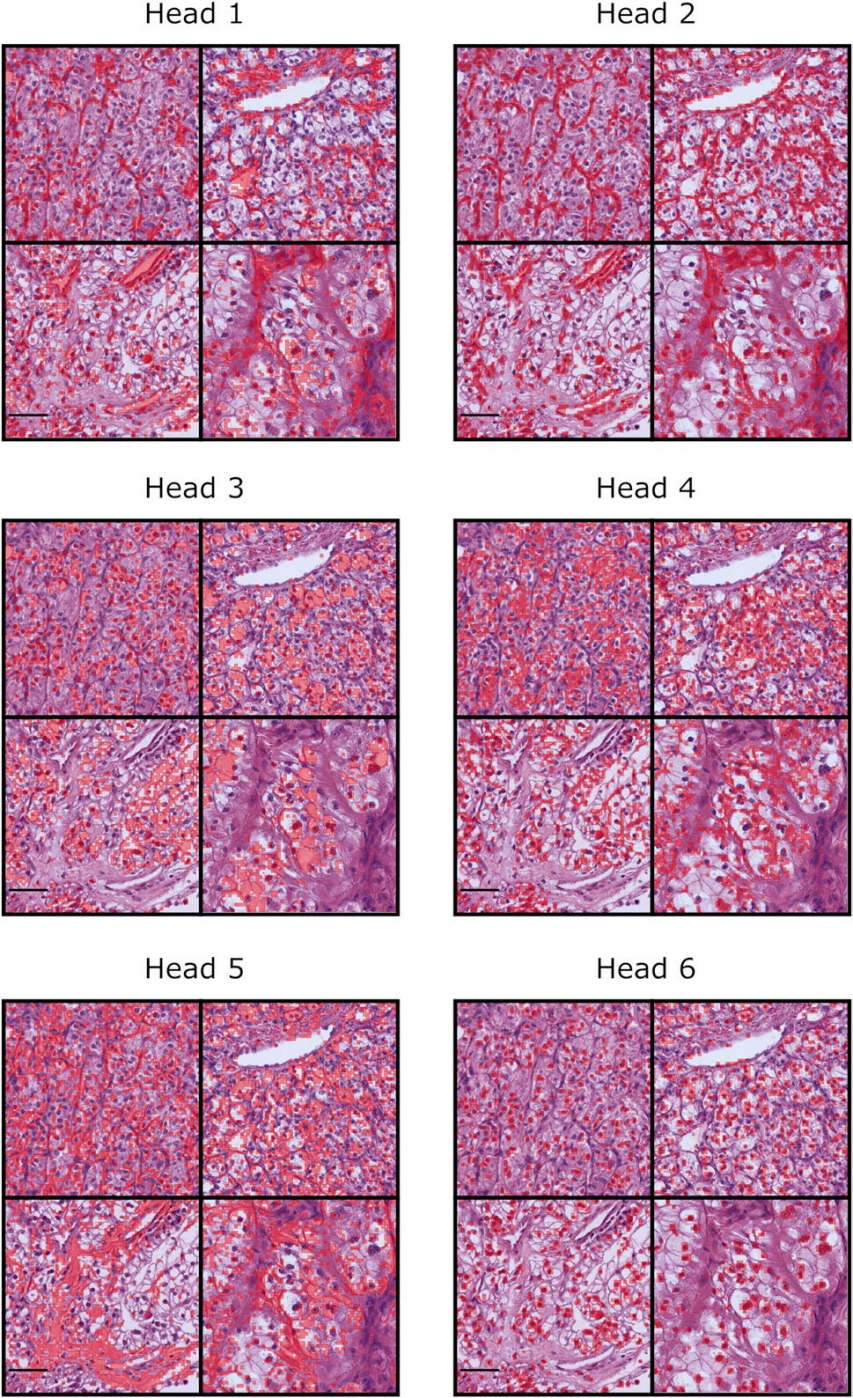
Visualisation of the DINO-ViT attention heads. The six different attention heads which emerged from the training process of the DINO-ViT model are shown. For each attention head, image sections of four different patient slides are shown. The respective structure that the model identified recurrently and assessed in the attention head is coloured red. Scale bar = 50 µm

Heads 1, 2, and 5 mainly focused on the peritumoural stroma, heads 3 and 4 highlighted the cytoplasm (Fig. 3). Head 3 seemed to focus more on the clear cell, whereas head 4 more on the stained cytoplasm. Head 6 clearly focused on the cell nuclei. The recurring identification of these structures was quite evident and uniform for all four slides demonstrating the capability of the DINO-ViT in identifying recurrent structures within histological images.

## Discussion

In this work, we investigated the combination of a ViT, self-supervised learning using DINO and a LASSO-Cox regression analysis to predict survival from H&E-stained histological tumour sections of ccRCC. A significant uni- and multivariable risk stratification was achieved with the training set. The prediction of DSS also remained significant in multivariable analysis in the validation cohort.

The DINO-ViT architecture [11] was chosen mainly because this model learns recurring structures and image features independently of a pre-defined task. In principle, these features can, therefore, be used for many different tasks and a prediction model can be selected depending on the desired outcome. This approach provides robustness in out-of-distribution data. A similar approach was used by Chen et al. [16]. Here, extracted pre-defined image features were used in a diagnostic and a survival prediction model. The positive results of both studies demonstrate the multiplicity of tasks that can be performed with such architectures.

The positive results in univariable analysis show that the recurrent image features identified by DINO-ViT have prognostic relevance. Here, the question arises as to the extent at which the identified structures correspond to known pathological risk factors. Tumour size and grading have been shown to be significant prognostic factors for survival in RCC [17–19]. The results of multivariable analyses underline that our model can extract additional information from the histological tumour sections than can be achieved with the current pathological classifications, at least for the endpoint DSS. Even in subgroup analysis, including only patients with metastases, the model achieved a significant DSS and OS risk stratification in this high-risk population in the TCGA cohort, although it was not designed for this purpose and the threshold was not adapted. Probably due to the low number of patients, in the external validation cohort, there was no significant difference, although a trend for a meaningful stratification was seen for DSS. To develop a metastasis-specific model using this AI-architecture thus seems to be promising.

Visualisation of the ViTs attention heads demonstrated that the model identified structures similar to the traditional concept of pathological assessment [17]. The possible advantage may be that subtle differences in cell or nucleus size and shape might be captured that are not represented in the established classifications, such as grading G1-G4. Several studies indicate that automated grading in RCC may provide a prognostically more relevant grading than manual grading [20, 21]. It has also been shown that certain genetic changes can manifest themselves in different phenotypic expressions, for example leading to differences in the cytoplasm or the stroma in ccRCC [22]. It has already been shown that several mutations in different cancer types can be detected on the H&E slide by AI [23, 24]. Additionally, standardisation and lack of interobserver variability might improve the predictive ability.

DINO-ViT thus has the potential to contribute to improved cancer outcome prediction, for example as part of a multivariable prediction model by adding DINO-ViT to an existing model or developing a new, even completely AI-based multivariable prediction model.

### Similar works

Wulczyn et al. achieved similar results on the TCGA-KIRC cohort for predicting DSS using a convolutional neural network (CNN) designed to predict survival data [7]. A significant risk stratification into three risk groups was achieved which remained significant in multivariable analysis (HR 1.88; *p* < 0.01) in addition to the tumour stage (HR 3.20; *p* < 0.01) while age was not a significant predictor. Interestingly, four other tumour types across the TCGA cohorts showed significant DSS prediction with Wulczyns’s model architecture, while in five other cancer types, such as bladder cancer, no significant DSS prediction was achieved. Tabibu et al. developed a method in which shape features were extracted from RCC histopathology images and subsequently tested for their predictive value of OS [25]. In multivariable analysis, it remained a significant predictor (HR 2.27; *p* < 0.01). In contrast to work presented here, no external validation was performed in either study and thus, the robustness and generalisability on unseen data of these interesting architectures remains unclear. An externally validated method was developed by Chen et al. as described above [16]. The LASSO-Cox model showed a significant predictive ability in the validation cohort in multivariable analysis. In contrast to our study, however, the extracted image features were defined beforehand, while the DINO-ViT model has the potential to use image structures that have not yet been considered relevant. Additionally, the risk score threshold used for dividing the cohorts into low- and high-risk groups was calculated individually in the training and validation sets. In our study, the threshold was defined in the training set and this threshold was used for the external validation set since a cohort-specific threshold definition significantly increases the risk of overfitting and might overestimate external applicability.

### Limitations

The retrospective design of our study is the major limitation of this work. Additionally, a larger dataset is necessary to enhance the robustness of our algorithm. Also, the clinical data appears very robust in the prediction of survival, thus the benefit of adding an AI model is not certain in this regard and requires further evaluation. While the TCGA-KIRC cohort has proven very useful in recent years, it has some shortcomings in the clinical follow-up data. The follow-up time was shorter with a median of 37 months than in our cohort (108 months). A longer follow-up would have helped training the Cox hazard model more accurately. Additionally, for TCGA-KIRC data on DSS is incomplete and an established approximation of DSS had to be used. Again, more accurate data might have resulted in an even more robust model. However, the successful external validation in a cohort with precise information on DSS and a follow-up of sufficient length confirm the successful training and use of this model.

## Conclusion

Our externally validated DINO-ViT architecture provides elevated level of explainability and interpretability. Thus, if this model is improved further and our early results can be confirmed in a prospective evaluation, clinical implementation as an assessment tool to guide therapy or follow-up intervals might be feasible with the goal of advancing current clinicopathological paradigms.

## Data Availability

Relevant TCGA training set data can be provided upon request. Validation set data cannot be shared because it is protected by the data protection law. Data requests would require a decision on an individual basis and need to be approved by the local ethics committee.

## References

[1] Feng X, Zhang L, Tu W, Cang S (2019). Frequency, incidence and survival outcomes of clear cell renal cell carcinoma in the United States from 1973 to 2014: a SEER-based analysis. Medicine (Baltimore).

[2] Grimm J, Zeuschner P, Janssen M, Wagenpfeil S, Hartmann A, Stohr C (2019). Metastatic risk stratification of clear cell renal cell carcinoma patients based on genomic aberrations. Genes Chromosom Cancer.

[3] Sanjmyatav J, Matthes S, Muehr M, Sava D, Sternal M, Wunderlich H (2014). Identification of high-risk patients with clear cell renal cell carcinoma based on interphase-FISH. Br J Cancer.

[4] Nientiedt M, Muller K, Nitschke K, Erben P, Steidler A, Porubsky S (2021). B-MYB-p53-related relevant regulator for the progression of clear cell renal cell carcinoma. J Cancer Res Clin Oncol.

[5] Kather JN, Krisam J, Charoentong P, Luedde T, Herpel E, Weis CA (2019). Predicting survival from colorectal cancer histology slides using deep learning: a retrospective multicenter study. PLoS Med.

[6] Wulczyn E, Steiner DF, Moran M, Plass M, Reihs R, Tan F (2021). Interpretable survival prediction for colorectal cancer using deep learning. NPJ Digit Med.

[7] Wulczyn E, Steiner DF, Xu Z, Sadhwani A, Wang H, Flament-Auvigne I (2020). Deep learning-based survival prediction for multiple cancer types using histopathology images. PLoS ONE.

[8] Mondal AK, Bhattacharjee A, Singla P, Prathosh AP (2022). xViTCOS: explainable vision transformer based COVID-19 screening using radiography. IEEE J Transl Eng Health Med.

[9] Park S, Kim G, Oh Y, Seo JB, Lee SM, Kim JH (2022). Multi-task vision transformer using low-level chest X-ray feature corpus for COVID-19 diagnosis and severity quantification. Med Image Anal.

[10] Wu Y, Qi S, Sun Y, Xia S, Yao Y, Qian W (2021). A vision transformer for emphysema classification using CT images. Phys Med Biol.

[11] Caron M, Touvron H, Misra I, Jégou H, Mairal J, Bojanowski P et al (2021) Emerging properties in self-supervised vision transformers. arXiv preprint arXiv:210414294

[12] Collins GS, Reitsma JB, Altman DG, Moons KG (2015). Transparent reporting of a multivariable prediction model for individual prognosis or diagnosis (TRIPOD): the TRIPOD statement. Br J Cancer.

[13] Liu J, Lichtenberg T, Hoadley KA, Poisson LM, Lazar AJ, Cherniack AD (2018). An integrated TCGA pan-cancer clinical data resource to drive high-quality survival outcome analytics. Cell.

[14] Bankhead P, Loughrey MB, Fernández JA, Dombrowski Y, McArt DG, Dunne PD (2017). QuPath: open source software for digital pathology image analysis. Sci Rep.

[15] Macenko M, Niethammer M, Marron JS, Borland D, Woosley JT, Xiaojun G et al (eds) (2009) A method for normalizing histology slides for quantitative analysis. In: 2009 IEEE International Symposium on Biomedical Imaging: from Nano to Macro; 2009 28 June–1 July 2009

[16] Chen S, Zhang N, Jiang L, Gao F, Shao J, Wang T (2021). Clinical use of a machine learning histopathological image signature in diagnosis and survival prediction of clear cell renal cell carcinoma. Int J Cancer.

[17] Delahunt B, Cheville JC, Martignoni G, Humphrey PA, Magi-Galluzzi C, McKenney J (2013). The International Society of Urological Pathology (ISUP) grading system for renal cell carcinoma and other prognostic parameters. Am J Surg Pathol.

[18] Keegan KA, Schupp CW, Chamie K, Hellenthal NJ, Evans CP, Koppie TM (2012). Histopathology of surgically treated renal cell carcinoma: survival differences by subtype and stage. J Urol.

[19] Schiavina R, Borghesi M, Chessa F, Dababneh H, Bianchi L, Della Mora L (2015). The prognostic impact of tumor size on cancer-specific and overall survival among patients with pathologic T3a renal cell carcinoma. Clin Genitourin Cancer.

[20] Holdbrook DA, Singh M, Choudhury Y, Kalaw EM, Koh V, Tan HS (2018). Automated renal cancer grading using nuclear pleomorphic patterns. JCO Clin Cancer Inform.

[21] Tian K, Rubadue CA, Lin DI, Veta M, Pyle ME, Irshad H (2019). Automated clear cell renal carcinoma grade classification with prognostic significance. PLoS ONE.

[22] Chen YB, Mirsadraei L, Jayakumaran G, Al-Ahmadie HA, Fine SW, Gopalan A (2019). Somatic mutations of TSC2 or MTOR characterize a morphologically distinct subset of sporadic renal cell carcinoma with eosinophilic and vacuolated cytoplasm. Am J Surg Pathol.

[23] Kather JN, Heij LR, Grabsch HI, Loeffler C, Echle A, Muti HS (2020). Pan-cancer image-based detection of clinically actionable genetic alterations. Nat Cancer.

[24] Fu Y, Jung AW, Torne RV, Gonzalez S, Vöhringer H, Shmatko A (2020). Pan-cancer computational histopathology reveals mutations, tumor composition and prognosis. Nat Cancer.

[25] Tabibu S, Vinod PK, Jawahar CV (2019). Pan-renal cell carcinoma classification and survival prediction from histopathology images using deep learning. Sci Rep.

